# Evaluation of the mechanism of epithelial-mesenchymal transition in human ovarian cancer stem cells transfected with a WW domain-containing oxidoreductase gene

**DOI:** 10.3892/ol.2014.2063

**Published:** 2014-04-14

**Authors:** HONGCHAO YAN, YUPING SUN

**Affiliations:** 1 Department of Oncology, Shandong University School Of Medicine, Jinan, Shandong 250012, P.R. China; 2 Department of Medical Oncology, Jinan Central Hospital Affiliated to Shandong University, Jinan, Shandong 250013, P.R. China

**Keywords:** WW domain-containing oxidoreductase gene, epithelial-mesenchymal transition, ovarian cancer stem cells

## Abstract

The aim of the present study was to investigate the impact of the WW domain-containing oxidoreductase (*WWOX*) gene on the mechanisms underlying epithelial-mesenchymal transition (EMT) in human ovarian cancer stem cells. Western blot analysis was performed to detect the differences in the expression of the EMT markers, E-cadherin, β-catenin, N-cadherin, vimentin and fibronectin, between human ovarian cancer stem cells and the human epithelial ovarian carcinoma cell line, HO-8910. A pcDNA3.1-WWOX eukaryotic expression vector was subsequently transfected into the ovarian cancer stem cells (recombinant plasmid group) or an empty plasmid (empty plasmid group) and non-transfected ovarian cancer stem cells (blank control group) served as the controls. Following the transfection of the *WWOX* gene, methyl thiazolyl tetrazolium cell viability and Transwell^®^ invasion assays, and western blot analysis were performed to detect changes in the proliferative capability and invasive capacity of ovarian cancer stem cells, as well as the expression of EMT markers and regulatory factors, Elf5 and Snail. The expression levels of E-cadherin and β-catenin in the ovarian cancer stem cells were identified to be significantly lower than those in the HO-8910 cells, whereas the expression levels of N-cadherin, vimentin and fibronectin in the ovarian cancer stem cells were found to be significantly higher than those in the HO-8910 cells. At each time point, the cellular proliferative capacity of the recombinant plasmid group was observed to be significantly lower than that of the empty plasmid or blank control groups (P<0.05 vs. the controls). The number of penetrating cells in the recombinant plasmid, empty plasmid and the blank control groups were 105.5±3.1, 199.7±3.4 and 191.4±4.1, respectively (mean ± standard error of the mean; P<0.05 vs. the controls). In addition, the protein expression of E-cadherin, β-catenin and Elf5 in the recombinant plasmid group was found to be significantly higher than that in the other two groups, whereas the protein expression of N-cadherin, vimentin, fibronectin and Snail in the recombinant plasmid group was significantly lower than that in the other two groups. An EMT exists in ovarian cancer stem cells, and the *WWOX* gene inhibits the cellular proliferation of ovarian cancer stem cells and reduces their invasive capability. Therefore, the *WWOX* gene may reverse the EMT in ovarian cancer stem cells by regulating the expression of the EMT regulatory factors, Elf5 and Snail.

## Introduction

The early diagnosis of ovarian cancer is complex, and thus leads to ineffective treatment and the highest mortality rate of any gynecological malignancy, which poses a serious threat to the health of females. Detailed investigations of the biological behavior and mechanisms underlying ovarian cancer have been performed in order to identify an improved treatment for this gynecological cancer and recently, the promotion of the cancer stem-cell theory has provided a novel perspective for determining the biological behavior of ovarian cancer. Cancer stem cells possess a self-renewal capacity within the tumor, which enables unlimited cellular proliferation and differentiation. Therefore, cancer stem cells are considered to be the source of relapse, tumorigenesis, tumor invasion, tumor metastasis, cancer drug resistance and recurrence (1). Marked invasiveness and metastatic capacity are the predominant characteristics of cancer stem cells. In addition, the epithelial-mesenchymal transition (EMT) is important in cancer stem cell metastasis and recurrence (2). In the present study, the human epithelial ovarian carcinoma cell line, HO-8910 was analyzed, which was established from a patient with poorly differentiated ovarian papillary serous cystadenocarcinoma. The cells were grown in suspension culture with paclitaxel-combined serum-free medium (Hangzhou Sijiqing Biology Engineering Materials Co., Ltd., Hangzhou, China) in order to successfully screen the ovarian cancer stem cells for the expression of CD133^+^ and CD117^+^ *in vivo* and *in vitro,* prior to further identification of their specific markers and biological characteristics (3). Our previous study identified that the WW domain-containing oxidoreductase (*WWOX)* gene significantly affects the biological behavior of human ovarian cancer cells (4). In the current study, to further investigate the impact of the *WWOX* gene on ovarian cancer stem cells, the eukaryotic expression vector, pcDNA3.1-WWOX, was transfected into ovarian cancer stem cells to investigate the impact on EMT and its mechanism of action.

## Materials and methods

### Materials

Human ovarian cancer stem cells were screened and collected at Central Laboratory of Shandong University School of Medicine (Jinan, China). The pcDNA3.1-WWOX eukaryotic expression vector was also prepared and stored in this laboratory. The Lipofector^TM^ liposomal transfection reagent was provided by the Beyotime Institute of Biotechnology (Shanghai, China) and Transwell^®^ chambers were purchased from Chemicon (Billerica, MA, USA). The E-cadherin, β-catenin, vimentin, fibronectin, Elf5 and Snail primary antibodies were purchased from Sigma-Aldrich (St. Louis, MO, USA). This study was approved by the Ethics Committee of Shandong University (Jinan, China).

### Cell culture

The human ovarian cancer stem cells were subcultured using serum-free medium, and the HO-8910 cell line was incubated using RPMI-1640 medium (Hyclone, South Logan, UT, USA), in a thermostat-equipped, humidified incubator containing 5% CO_2_ at 37°C.

### Western blot analysis to detect the differential expression of EMT markers in ovarian cancer stem and HO-8910 cells

In total, two groups of cells were harvested during the log growth phase and incubated in 200 μl lysis buffer on ice. Total protein concentrations from cell lysates were determined using the bicinchoninic acid assay (Hangzhou Sijiqing Biology Engineering Materials Co., Ltd.). Next, the total protein was separated using 10% sodium dodecyl sulphate-polyacrylamide gel and the protein bands were transferred to a nitrocellulose membrane (Qiagen, Hilden, Germany). The nitrocellulose membranes were blocked with 5% non-fat milk for 60 min and incubated with the rabbit anti-human E-cadherin primary monoclonal antibody (1:1,000) at 4°C overnight. Following three 10 min washes with washing solution (Hangzhou Sijiqing Biology Engineering Materials Co., Ltd., Hangzhou, China), the horseradish peroxidase-conjugated goat anti-rabbit secondary antibody (1:10,000) was further incubated at room temperature for 2 h. An enhanced chemiluminescence reagent (Hangzhou Sijiqing Biology Engineering Materials Co., Ltd.) was used to visualize the protein blots on highly sensitive X-ray film (Shanghai Shenggong Biological Engineering Co., Ltd., Shanghai, China) following exposure and development in the dark. The detection of β-catenin, N-cadherin, vimentin and fibronectin was performed according to the aforementioned procedures.

### Gene transfection and experimental groups

The lipofection technique was used to transfect the eukaryotic expression vector carrying the *WWOX* gene into the ovarian cancer stem cells (recombinant plasmid group) according to the manufacturer’s instructions for the Lipofector^TM^ liposomal transfection reagent. The stably transfected cells were selected and further cultured, while the empty plasmid (empty plasmid group) and non-transfected ovarian cancer stem cells (blank control group) served as the controls.

### Reverse transcription-polymerase chain reaction (RT-PCR) for detection of WWOX mRNA expression

A total of three groups of cells was harvested during the log growth phase and total RNA was extracted using TRIzol reagent (Chemicon, Temecula, CA, USA). The standard conditions for RT-PCR were followed according to the manufacturer’s instructions for the reverse-transcription reagent (Promega Corporation, Madison, WI, USA). The primer sequences used were as follows: Forward, 5′-CACGCATTTTAGAAGAATGG-3′ and reverse, 5′-GACAGCAGCACAGTACACG-3′ (amplified fragment size of 598 bp) for *WWOX*; and forward, 5′-CGGGAAGCTTGTGATCAATGG-3′ and reverse, 5′-GGCAGTGATGGCATGGACTG-3′ (amplified fragment size of 357 bp) for the housekeeping gene, glyceraldehyde-3-phosphate dehydrogenase (*GAPDH*). The RT-PCR reaction conditions used were as follows: 30 Cycles of 94°C for 45 sec, 55°C for 60 sec; 72°C for 60 sec; and a 72°C primer extension for 10 min. The PCR-amplified cDNA fragments were detected using 2% agarose gel electrophoresis and observed under ultraviolet illumination using an image capture system (4100, Olympus, Tokyo, Japan). The cells expressing the *WWOX* gene exhibited an amplified band of 598 bp, whereas cells without the *WWOX* gene did not exhibit a specific amplified band. Finally, the automatic analyzer ChemiImager 5500 imaging software (Alpha Innotech Corp., San Diego, CA, USA) was used to quantify the amplified bands using the *WWOX*/*GAPDH* content ratio to measure the relative expression levels of *WWOX* mRNA.

### Methyl thiazolyl tetrazolium (MTT) assay for detection of ovarian cancer stem cell proliferation

The cells were grouped as aforementioned and the three groups of cells were seeded in 96-well plates (Hangzhou Sijiqing Biology Engineering Materials Co., Ltd.) at a density of 1.5×10^4^ cells/well and incubated for various time periods (one, two, three, four, five or six days). A total of 20 μl MTT working solution was added at the end of each time point and incubated in a CO_2_ incubator at 37°C for an additional 4 h; dimethyl sulfoxide was added to terminate the reaction. The reaction product was measured in each well at an absorbance (A) value of 490 nm using an ELISA plate reader (Elx910, Qiagen) and the corresponding cellular growth curves were plotted.

### Analysis of cancer invasion by ovarian cancer stem cells in vitro using the Transwell^®^ cell migration/invasion Matrigel assay

A precoated Matrigel insert was placed between the upper and lower invasion chambers and 200 μl of a HO-8910 single-cell suspension (containing ~1×10^5^ cells) was plated in the invasion insert and incubated in a CO_2_ incubator at 37°C for 12 h. The non-invasive cells and Matrigel medium were subsequently removed from the insert. Next, the insert was fixed and stained with hematoxylin and eosin to visualize the invasive cells. The number of invasive cells was measured under a light microscope (CKX41, Olympus) and each group of cells was assessed in the three individual inserts, and in triplicate.

### Western blot analysis to detect the expression of EMT markers and regulatory factors, Elf5 and Snail, in ovarian cancer stem cells

The experiment was divided into three groups: the recombinant plasmid group, empty plasmid group and the blank control group. Western blot analysis was performed using the same method as described previously for the detection of the differential expression of EMT markers in ovarian cancer stem and HO-8910 cells

## Results

### Expression of EMT markers in ovarian cancer stem and HO-8910 cells

**Figure 1:**
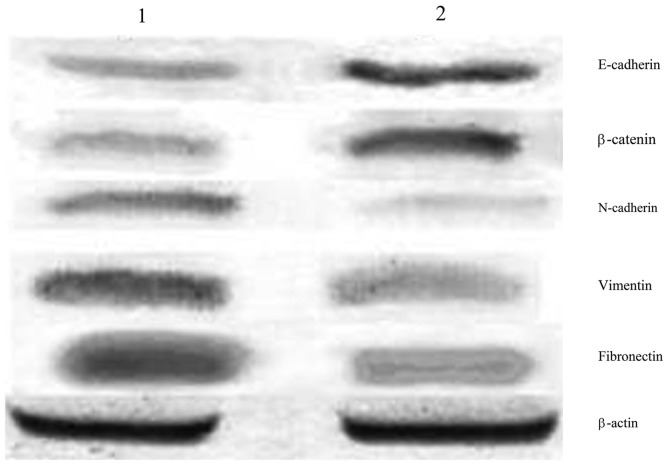
Differences among epithelial-mesenchymal transition markers in ovarian cancer cells as detected by western blot analysis. Lane 1, stem cells; and lane 2, human epithelial ovarian carcinoma HO-8910 cells.

The protein expression of E-cadherin and β-catenin in ovarian cancer stem cells, as detected by western blot analysis, was 0.294±0.023 and 0.313±0.017, respectively; significantly lower than that in the HO-8910 cells (0.771±0.031 for E-cadherin and 0.752±0.011 for β-catenin; P<0.05). Conversely, the expression of N-cadherin, vimentin and fibronectin in ovarian cancer stem cells was 0.698±0.012, 0.839±0.021 and 0.847±0.022, respectively; significantly higher than that in HO-8910 cells (0.228±0.022 for N-cadherin, 0.353±0.027 for vimentin and 0.322±0.019 for fibronectin; P<0.05; Fig. 1).

### Differences in WWOX mRNA expression following the transfection of ovarian cancer stem cells with the WWOX gene

**Figure 2:**
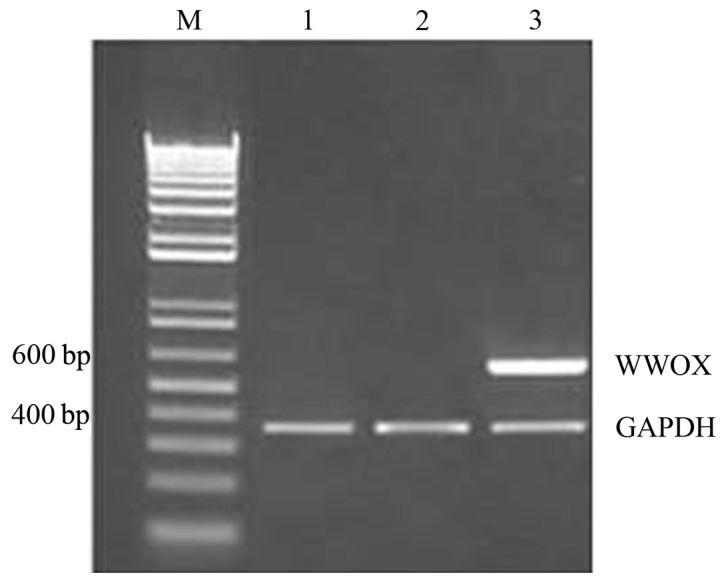
Detection of WWOX mRNA expression using reverse transcription-polymerase chain reaction. Lane 1, blank control; lane 2, empty plasmid; and lane 3, recombinant plasmid. WWOX, WW domain-containing oxidoreductase; GAPDH, glyceraldehyde-3-phosphate dehydrogenase.

The results of the RT-PCR analysis demonstrated that *WWOX* mRNA expression in the recombinant plasmid group was high, however, *WWOX* mRNA was not detected in the empty plasmid or blank control groups (Fig. 2).

### Changes in cell proliferation following the transfection of ovarian cancer stem cells with the WWOX gene

**Figure 3:**
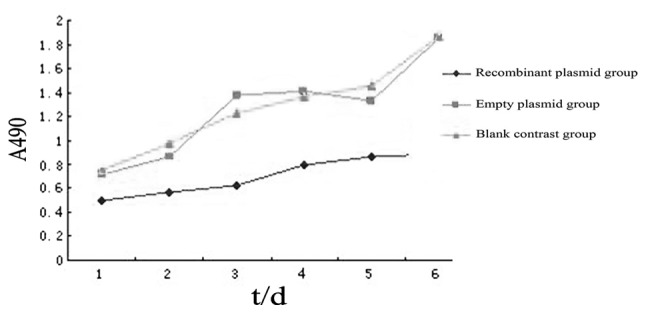
Growth curve of ovarian cancer stem cells following transfection with pcDNA3.1-WW domain-containing oxidoreductase. A490, absorbance at 490 nm. t/d, time (days).

The MTT assay revealed that the A values of the recombinant plasmid group following one, two, three, four, five and six days of incubation were 0.502±0.004, 0.567±0.011, 0.622±0.016, 0.798±0.002, 0.861±0.022 and 0.892±0.013, respectively; significantly lower than that of the blank control or empty plasmid groups at the corresponding time points (P<0.05). No statistically significant differences were identified between the empty plasmid and blank control groups (P>0.05; Fig. 3).

### Changes in invasive capacity following the transfection of ovarian cancer stem cells with the WWOX gene

*In vitro* invasion assays using the Transwell^®^ chamber detected the following number of invasive cells in the recombinant plasmid, empty plasmid and blank control groups: 105.5±3.1, 199.7±3.4 and 191.4±4.1, respectively. Statistically significant differences were identified between the recombinant plasmid and control groups (P<0.05), however, no statistically significant differences were identified between the empty plasmid and blank control groups (P>0.05).

### Changes in EMT markers and regulatory factors following the transfection of ovarian cancer stem cells with the WWOX gene

**Figure 4:**
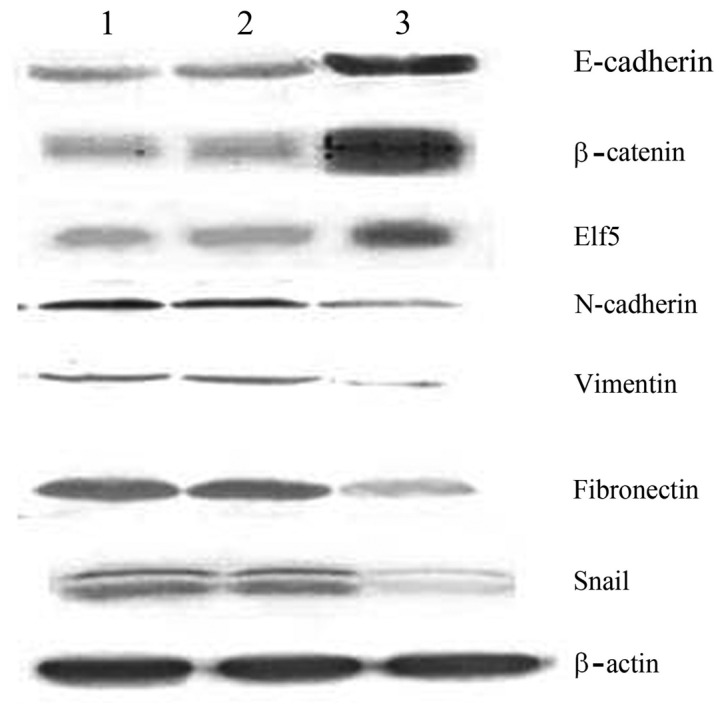
Differences among epithelial-mesenchymal transition markers and regulatory factors in three groups of cells as detected by western blot analysis. Lane 1, blank control; lane 2, empty plasmid; and lane 3, recombinant plasmid.

The results of western blot analysis revealed that the expression levels of E-cadherin, β-catenin and Elf5 in the recombinant plasmid group were 0.762±0.007, 0.911±0.016 and 0.841±0.021, respectively; significantly higher than those of the control groups (P<0.05). In addition, the expression levels of N-cadherin, vimentin, fibronectin and Snail in the recombinant plasmid group were 0.212±0.008, 0.136±0.017, 0.311±0.015 and 0.339±0.027, respectively; significantly lower than those of the control groups (P<0.05; Fig. 4).

## Discussion

The EMT occurs in epithelial cells under specific physiological or pathological conditions. EMT is a process in which unique characteristics of certain mesenchymal cells are acquired, including epithelial cell polarity, intracellular adhesion and loss of specific cell surface markers. Cytoskeletal remodeling occurs in these cells and the cells subsequently obtain a mesenchymal-like phenotype. These changes increase the invasive capacity of tumor cells and enhance their degree of malignancy (5). The major molecular characteristics of EMT are the downregulation of the epithelial cell markers, E-cadherin and β-catenin, and upregulation of the markers of mesenchymal phenotype, vimentin, fibronectin and N-cadherin. The downregulation of E-cadherin indicates EMT and is the prerequisite for epithelial tumor cell invasion (6,7).

E-cadherin, as the most significant EMT marker, is predominantly affected by the regulation of the transcription factor, Snail. Snail appears to be a basic helix-loop-helix transcription factor in *Drosophila*, rodents and humans, which belongs to the family of zinc-finger proteins. Snail binds to the E-cadherin promoter to inhibit the transcription of E-cadherin and subsequently causes the marked upregulation of N-cadherin, vimentin and fibronectin, which is a hallmark of EMT (8). Snail, as a transcription factor at the center of the signaling cascade, is regulated by the upstream transcription factor, Elf5 (9). Elf5 is a transcription factor present in mammals that significantly inhibits breast cancer and certain hormone-related tumors. Elf5 may directly inhibit the expression of the Snail transcription factor to further suppress the EMT and thereby reduce the invasiveness of breast cancer cells (10).

In 2000, the *WWOX* gene was isolated and identified as a tumor suppressor gene by Bednarek *et al (*11*)* using shotgun sequencing technology. Furthermore, the *WWOX* gene is mapped to the human chromosome 16q23.3–24.1, which covers the entire chromosomal fragile site, FRA16D. The WWOX peptide contains 414 amino acids with two WW domains at the N-terminal. WW functional domains are associated with protein-protein interactions, which are necessary for tumor inhibition by tumor suppressor genes through various signal-transduction pathways. Our previous studies confirmed that the *WWOX* gene is regulated by genetic and epigenetic mechanisms (12–17). In order to further investigate the impact of *WWOX* genes on ovarian cancer stem cells, the current study selected human ovarian cancer stem cells and the human epithelial ovarian carcinoma cell line, HO-8910 as experimental models. Western blot analyses were used to detect the differences in EMT markers, including the expression of E-cadherin, β-catenin, N-cadherin, vimentin and fibronectin, in the two groups. The results revealed that the expression of E-cadherin and β-catenin in ovarian cancer stem cells was significantly lower than that in the HO-8910 cancer cell line, whereas the expression of N-cadherin, vimentin and fibronectin in ovarian cancer stem cells was significantly higher than that in the HO-8910 cells. This indicated that the EMT phenomenon occurs in ovarian cancer stem cells. The ovarian cancer stem cells were transfected with the pcDNA3.1-WWOX and pcDNA3.1 eukaryotic expression vectors and the cells were partitioned into recombinant plasmid, empty plasmid and blank control groups, according to the plasmid characteristics. The three cell groups were subsequently tested by MTT assay to measure the cell proliferation rates, a Transwell^®^ invasion assay to determine the invasive capacities and western blot analysis to detect the changes in EMT protein marker expression levels, as well as expression of EMT regulatory factors, Elf5 and Snail. The results established that the *WWOX* gene inhibits ovarian stem cell proliferation and reduces its invasive capacity following transfection. In addition, *WWOX* was found to significantly upregulate E-cadherin, β-catenin and Elf5, whilst significantly downregulating N-cadherin, vimentin, fibronectin and Snail. In conclusion, these results indicated that the *WWOX* gene reverses the EMT phenomenon in ovarian cancer stem cells by regulating the expression of various transcription factors and reduces tumor invasion, providing a potential novel therapeutic target for ovarian cancer.
